# Editorial

**Published:** 1841-05-08

**Authors:** 


THE MEDICAL EXAMINER.

PHILADELPHIA, MAY 8, 1841.

### EPIDEMIC OF SMALL POX.

For some years past, small pox has been on the increase in London and other parts of Great Britain. It appears generally as an epidemic, lasts a few months, and disappears, obeying, in this respect, as ascertained by Mr. Farr, considerable regularity in its character and period of return. The disease was especially prevalent during the last autumn in London, and about the beginning of the present year showed itself at Philadelphia, and at most of the other ports. It is now declining, after lasting a few months, and was marked throughout its course by some peculiar features. At first it was confined to the filthiest allies of the town, and prevailed chiefly amongst the blacks; but after the epidemic had lasted for some time, several cases occurred in different parts of the city, attended with extraordinary malignity, and proved fatal. These took place in that class of persons whose circumstances were the most comfortable, and who were least liable to come in direct contact with the disorder. One case of small pox, and another of varioloid, occurred in the Philadelphia Hospital among the lunatics; the patient attacked with small pox had not left the institution for years, and could by no possibility come into actual communication with persons affected; this was the first which occurred. The fact itself was singular, as it is not believed that any case had occurred in the institution for many years, although the average population of almshouse and hospital is about twelve hundred.

Few as have been the sufferers from these epidemics, they were living under such different circumstances, and at such remote distances from each other, that the cause of the disease was evidently a widely spread one, but fortunately did not last very long, or acquire a high degree of intensity.

As might be expected, much anxiety was manifested on the subject of re-vaccination. Some physicians have contemned the practice as useless and troublesome, but the large majority have certainly decided in its favour. There is this practical reasoning on the subject, vaccination does not always succeed when the physician is pretty well satisfied with the appearance of the pustule, and re-vaccination gives another chance to these doubtful cases. This way of viewing the subject does not, of course, determine the question as to the wearing out or continuance of the protection, nor even whether the protection of vaccination is sufficient or not. It simply gives an additional chance of success, and that surely constitutes a satisfactory reason for resorting to it—at least in those cases in which it is desired by the patient.

---

In our next number will appear an account of the case of the late President.

